# Review: Piglets’ (Re)Feeding Patterns, Mineral Metabolism, and Their Twisty Tail

**DOI:** 10.3390/metabo15070480

**Published:** 2025-07-16

**Authors:** Theo van Kempen, Eugeni Roura

**Affiliations:** 1 Animal Science Department, North Carolina State University, Raleigh, NC 27695-7621, USA; 2 Nutrition and Chemosensory Group, Centre for Animal Science and Centre for Nutrition and Food Sciences, Queensland Alliance for Agriculture and Food Innovation, The University of Queensland, St Lucia, QLD 4072, Australia; e.roura@uq.edu.au

**Keywords:** hypophosphatemia, post-weaning edema, refeeding syndrome, swine, tail-biting

## Abstract

The appearance rate of nutrients into systemic circulation affects hormones like insulin and through that efficiency of growth. This also affects mineral requirements critical for metabolism, notably phosphate (P), magnesium (Mg), and potassium (K). Fasting animals have a downregulated metabolism, upon which P, Mg, and K are exported from their cells into the blood and are subsequently excreted in their urine. Abrupt resumption of feed intake, especially of highly glycemic feeds, creates an acute need for these minerals, which can result in deficiency symptoms, particularly with P deficiency. In human medicine, this is called refeeding syndrome: a large meal after a period of fasting can prove fatal. Young animals seem to be especially sensitive, likely driven by their ability to grow rapidly and thus to drastically upregulate their metabolism in response to insulin. Symptoms of P deficiency are fairly a-specific and, consequently, not often recognized. They include edema, which makes it appear as if piglets are growing well, explaining the high gain/feed rate typically seen immediately after weaning, even when piglets are eating at or below the maintenance requirements. Phosphate deficiency can also result in hypoxia and hypercarbia, which may trigger ear necrosis, *Streptococcus suis* infections, or even death. Hypophosphatemia can also trigger rhabdomyolysis, which may contribute to tail-biting, but this requires further study. Arguably, when fasting cannot be avoided, diets for newly weaned piglets should be formulated to avoid these problems by lowering their glycemic load and by formulating diets according to the piglets’ actual requirements inspired by their genuine intake and health and not simply by extrapolating from older animals.

## 1. Introduction

The digestibility of nutrients is an important issue in animal nutrition, so it is subjected to very detailed evaluation by researchers [1]. In more recent years, studies on the digestion kinetics of starch and protein have entered the scene. Animal data, for instance, have revealed that starch digestion kinetics can affect feed intake and feed efficiency [2]. The rate of digestion may also affect hormones like insulin: a large meal with rapidly digestible starches resulted in strong peaks in blood insulin, one of the most important hormones managing the metabolism [3]. Even a moderate increase in insulin stimulates the use of glucose as an energy source by skeletal muscles. Moreover, it stimulates amino acid uptake and their incorporation into muscle proteins. Thus, insulin and growth hormones are key drivers of lean tissue growth. However, high levels of insulin stimulate the uptake of glucose for fat accretion by the adipose tissue, an undesirable phenomenon when out of control [4].

Not only feed composition but also feeding patterns affect hormones like insulin and, thus, growth, adiposity (body condition), and, consequently, feed efficiency. This pattern, provided the pig is eating *ad libitum*, is remarkably similar across life phases. Nursery, grow-finished pigs, and sows eat a large morning meal followed by a very large evening meal [5]. Patterns of hormones like melatonin and cortisol have diurnal patterns [6], and even insulin responses are affected by the time of the day [7]. It appears that the pig tolerates very high levels of insulin at dinner time without strongly triggering adiposity, while a similar meal at odd times can trigger adiposity [8].

## 2. Influence of Digestion Kinetics and Eating Patterns on Minerals

A topic that receives little attention today in nutrition is the influence of digestion kinetics and eating patterns on minerals. Insulin can stimulate protein and lipid syntheses, which require amino acids and energy, mainly in the form of glucose, as well as minerals and vitamins [4]. In this context, the most critical minerals are K, Mg, and P [9]. Cells acquire these minerals from the bloodstream when their metabolism is stimulated by insulin and export them back into the bloodstream when metabolism is downregulated. Phosphate is the backbone of energy metabolism in the cell; for example, adenosine monophosphate is phosphorylated to generate adenosine diphosphate and, subsequently, adenosine triphosphate (ATP). Phosphate is used to activate enzymes but also glucose and amino acids, allowing its use as an energy source or building block [4]. Magnesium is essential for the stabilization of ATP and as a cofactor for many enzymes [10]. Modern commercial swine diets usually already contain excesses of K and Mg from the feedstuffs used. In contrast, dietary P is administered as close to the required levels as possible. In addition, the P requirement for newly weaned piglets appears to be extrapolated from older animals [11].

Relevant to this issue, there is a possibility that sudden infant death syndrome (SIDS) could be caused by blood levels of P reaching a nadir in the early morning, aggravated by seasonal variation, where levels are typically lower in late winter and early spring. Phosphate, via 2, 3-bisphosphoglycerate (BPG), regulates oxygen release from red blood cells; a P shortage can therefore lead to low blood oxygen levels and, thus, suffocation, despite free access to air [12]. Interestingly, Lavoue et al. [13] investigated if SIDS was a phenomenon that also occurred in piglets; they concluded that weaned piglets can also die as a result of similar symptoms to those found with SIDS and with an incidence comparable to the incidence of SIDS in humans. Scrutiny of SIDS cases in infants revealed that in most cases, an event occurred in the weeks before SIDS was diagnosed that led to a disruption in food intake [14,15]. The triggering event can be, for example, a brief illness or a stressful episode, such as going to daycare for the first time.

## 3. Feed and Water Intake of Nursery Piglets

Weaning stress has been a key focus of the estimated 2000 trials run by or collaborated on by van Kempen while working in the industry, the vast majority of which have never published. Most of those experiments involved 500–1000 piglets housed in pens of 15 to 20 animals, in some cases equipped with feeding stations that allowed for tracking of the feed intake of individual animals. Some physiology-focused experiments were carried out with individually housed piglets. The diets administered were typically high-quality, complex nursery diets. Some of the observations reflected in this review were obtained during experiments that remained largely unpublished and are referred to as such in the text (unpublished results).

Feed intake disruptions, like those experienced by stressed infants, are common in pig production such as in the post-weaning phase. Unless special care is taken, newly weaned piglets may undergo several hours or even days of fasting. In addition, feed intake upon resumption is often erratic. Lessons drawn from a large number of unpublished studies show that during lactation, milk supply is ample, and, consequently, few pigs actively look for alternative sources of nutrition. Indeed, when creep feed is provided, its intake is dwarfed by milk intake. Individual pigs, though, may show very different eating patterns. Piglets that have difficulty nursing for one reason or another may become avid creep-eaters, and they are more likely to resume eating quickly after weaning. In contrast, pigs that are actively nursing do not know where to look for food when weaned. The closest thing to food from their perspective may well be drinking water. Water is provided from a nipple-like device, similar to milk, and, in the short term, it fills their bellies. Indeed, field observations of water intake show absurdly high water disappearance rates, typically peaking the day after weaning and ‘normalizing’ in the days after. Disappearance rates of 1 to 2 L per day seem common, with individual pigs drinking notably more [16]. The published lethal dose (LD50) for water is 90 g/kg BW for humans [17]. Extrapolating to a piglet weighing 7 kg, that equates to 0.8 L of water. Thus, piglets, on average, consume (or eliminate) well over the human LD50 for water. Interestingly, Schloerb et al. [18] exposed the jejunum to pure water, which resulted in ruptured enterocytes, implying that excessive water can harm gut health. Efforts to correlate early water intake to performance over the entire nursery period, which was taken as a measure of health, however, failed.

Once the piglet realizes that water is not the food that it needs, it will explore its environment for alternative food sources. If a new diet is presented to the piglet, then it will typically only sample a small portion of this food. Although it is often overlooked in trials, when feed disappearance is carefully tracked, small quantities of feed (grams) disappear, typically during ‘dinner’ time on the day of weaning [16]. Why would only small quantities disappear when the piglet is hungry? Presumably, this is ‘early sampling’ or ‘food neophobia’ [19,20]; the piglet needs to know if the food is safe to eat. Animals in the wild are known to do this. If, after consuming a small quantity, nothing undesirable happens in the next one to two days (in line with the observations of Roura [21]), the animal assumes that the food is safe and, subsequently, it will start consuming the food in larger quantities, often with erratic intake patterns. Trials with electronic feeding stations have demonstrated this nicely [16]. Given all the stresses the piglet endures after weaning, though, some animals may associate the eating of a few grams of feed with an adverse health effect, and those animals refuse to eat for much longer than others (unpublished data). Interestingly, there is a rescue mechanism: animals that see others eat are more inclined to consider this food as safe (in line with the observations of Roura and Navarro [20]). Thus, the odd piglet that was already eating creep before weaning and happily continues to eat the same creep feed after weaning can stimulate others to deem that food as safe and eat it. This also illustrates that changing diets should not be timed with other stressful events.

Diet changes and interruptions in feed intake also happen at other events during the life of the piglet. Birth is actually another prime example, but fortunately, newborn piglets has been programmed to look for colostrum expediently after birth. In addition, newborn piglets have ample glycogen stores. Later in life, diet or housing changes but also failures in feed supply can hurt feed intake for various lengths of time. An interesting question is how the sow responds to relocation to the farrowing room and a low feed intake until farrowing (as compared to ad lib), followed by poor feed intake during farrowing, all while the metabolic demands on her are at an all-time high.

## 4. Refeeding Syndrome

**Figure 1:**
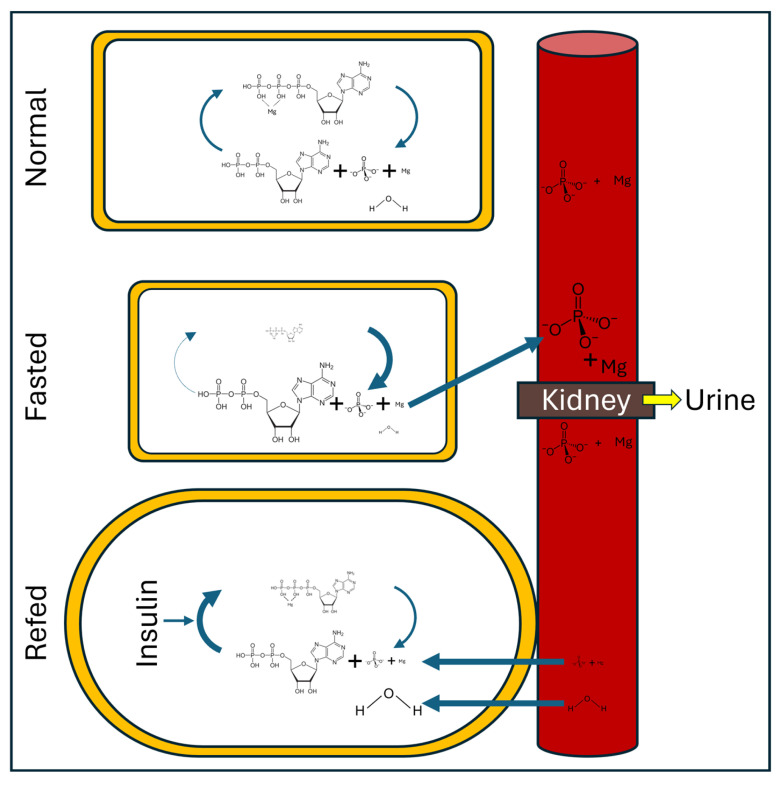
The influence of feed intake on the exchange of phosphate (P) and magnesium (Mg) between cells and the bloodstream. See text for details.

Fasting results in an interruption to nutrient supply when exogenous amino acid and energy supply effectively stops. With that, insulin and growth hormone secretion decline. Consequently, metabolic processes that are linked to growth decline. Within the muscle cells, there are also a myriad of changes. Enzymes involved in metabolism and protein synthesis are downregulated involving dephosphorylation and P is released in the process. ATP and phosphocreatine are only partially replenished after they have been used as energy carriers, resulting again in P, but also Mg, release. As the cell tries to maintain homeostasis, it exports these minerals, together with K, into the bloodstream (resulting in a small but transient increase in blood levels; Figure 1). Interestingly, there are no cellular or tissue storage systems for these minerals during times of energy shortages. Consequently, the animal stops re-absorbing them at the kidney level, and large amounts of P, Mg, and K appear in the urine. Technically, all this is simply setting a new steady state, and at this point, there are no deficiencies of these minerals [9,22].

The problem arises when the animal starts to eat again abruptly. A large meal following a period of fasting results in strong insulin secretion and, consequently, an important stimulation of metabolism. This means that cells want to fire up their metabolic activity for which large amounts of P, Mg, and K are needed. Hence, they pull this from the blood (Figure 1). The blood’s supply of these minerals, though, is very limited and, consequently, hypophosphatemia, hypomagnesemia, and hypokalemia can develop quickly [9,22]. Our findings suggest that hypophosphatemia is the most prevalent in piglets, but there may be conditions where Mg or K become first-limiting.

This health problem in human medicine is known as refeeding syndrome [9,22]. The phenomenon was first described when prisoners of war were liberated and offered a festive meal to celebrate their release; people survived the gruesome prison camps, but not the festive meal meant to celebrate their rescue [23,24]. In adult humans, it takes weeks of fasting to become prone to refeeding syndrome. In young animals, though, it appears that refeeding syndrome can develop much quicker, likely because of their high metabolic rate. In an infant and especially in a young piglet (capable of eating at 5 times maintenance), there is a very high growth potential provided there is adequate nutrient availability. This growth is a metabolically intensive process requiring large amounts of the minerals involved.

Results supporting the existence of refeeding syndrome in pigs have been published in a review [25]. In brief, quantification of urinary losses indeed showed that newly weaned pigs lose large amounts of P, Mg, and K in their urine in the first week after weaning. For example, on the day after weaning, piglets, on average, lost 12 mmol of P. This number is remarkable considering that plasma contained approximately 2.0 to 2.5 mM of P prior to weaning.

Analysis of blood levels of P in newly weaned pigs performed in research and field conditions in Europe, North America, and South America also revealed strong drops in plasma P levels that persisted for weeks after weaning, confirming that this is a common problem, not an isolated issue. For example, in a European trial, P increased immediately after weaning by approximately 13%, presumably due to the transfer from intra- to extracellular pools. However, subsequently, it dropped to less than half the preweaning levels on day 14. The lowest value recorded was 0.85 mM, which is severely deficient. Tumbleson and Kalish [26] report 2.8 ± 0.1 mM with a range of 2.1 to 3.3 as normal. Nadirs (lowest values) in P occur typically 1 to 2 weeks after weaning (which is in line with SIDS developing in infants in the weeks after an event that upsets their food intake).

**Figure 2:**
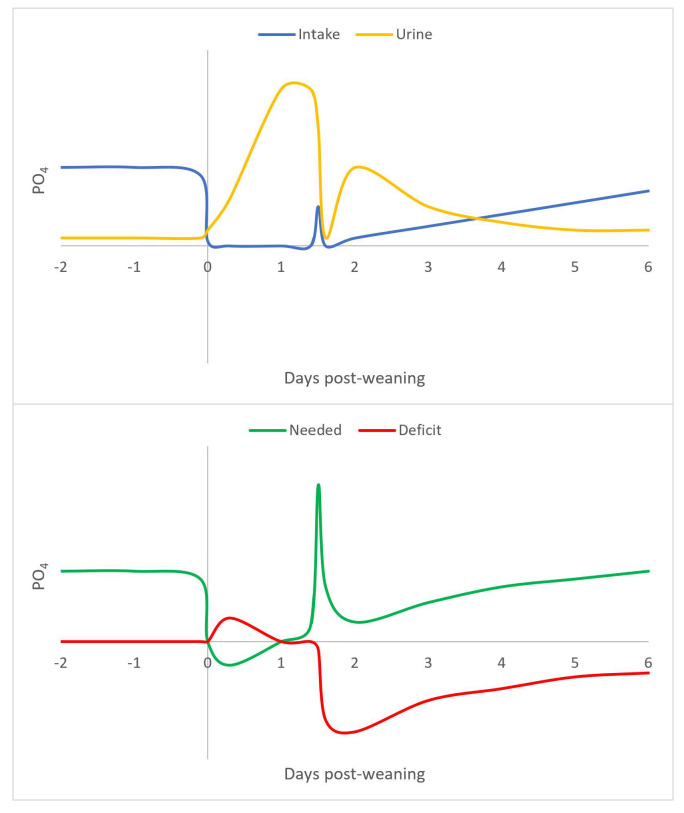
Theoretical representation of phosphate metabolism as affected by weaning. This figure is based on our own research.

Figure 2 provides a theoretical summary of P metabolism (but ignoring diurnal patterns). At the time of weaning, P intake drops to zero as animals are effectively fasting. This fasting results in a downregulation of metabolism, which results in the liberation of P from intracellular pathways (e.g., ATP), resulting in dramatic increases in urine P excretion (and also creatinine from the breakdown of creatine phosphate). If animals resume food intake erratically (hence the spike in P intake 1.5 days after weaning), the metabolism is triggered by insulin, resulting in a strong demand for P by cells. This demand, however, far exceeds the amount of P carried in the bloodstream, thus, resulting in hypophosphatemia. This hypophosphatemia is so severe that it takes an extended period to resolve. Thiamin has also been implicated in refeeding syndrome in humans [9]. In piglets, however, thiamin did not appear to be an issue (unpublished data), possibly because diets are routinely supplemented.

## 5. Hypophosphatemia

**Figure 3:**
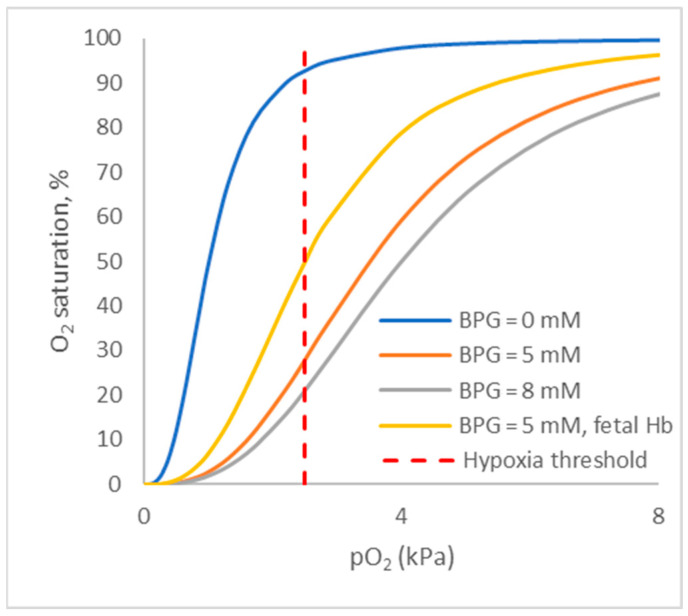
Hemoglobin (Hb) dissociation curve as affected by the concentration of 2, 3-bisphosphoglycerate (BPG; modeled after Hill [29]). O_2_ saturation is the percentage of oxygen-binding sites which are occupied on hemoglobin. pO_2_ is the partial oxygen pressure and is thus a measure of the amount of oxygen available to the tissue.

Phosphate deficiency is tricky to diagnose (and often ignored) from a clinical perspective. First, it is hard to detect through laboratory tests as blood P is not a measure of tissue adequacy per se. Second, symptoms are fairly non-specific. Long-term P shortage affects the bones. Acute shortages, though, can affect just about anything. Textbook symptoms in humans include anorexia, anemia, muscle weakness, skeletal defects, increased risks of infection, paresthesia, ataxia, and confusion [27]. One interesting phenomenon linked to hypophosphatemia is a decrease in the oxygenation of tissues. The mechanism behind this is that oxygen release from red blood cells is regulated by BPG which can generate a conformational change in hemoglobin, spurring oxygen release. Tissues in need of oxygen stimulate the production of BPG, a mechanism that is hindered when there is a P shortage. This effect is well known in respiration physiology and is typically referred to as the Bohr effect (after Bohr et al. [28]). Figure 3, modeled after Hill [29], demonstrates the impact of BPG on the amount of oxygen that stays bound to hemoglobin at various oxygen concentrations. At a tissue oxygen concentration of 4 kPa, in the absence of BPG, a maximum of 2% of the oxygen present in hemoglobin is released, but in the presence of 8 mM BPG, this is 50% (or 25 times more). In the absence of BPG, the tissue oxygen level needs to drop to 1 kPa for hemoglobin to release 50% of its oxygen (numbers are indicative only, as factors like pH and pCO_2_ play critical roles as well). Thus, when BPG formation is impeded, tissues can become hypoxic; theoretically, the animal can suffocate despite having free access to air. For reference, the hypoxia threshold for the brain is 2.5 kPa [30]. It should be noted that the data above are for adult (human) hemoglobin. Fetal hemoglobin apparently has a lower binding affinity for BPG [31]; therefore, the problem may potentially be more severe early in life when fetal hemoglobin is still present (Figure 3).

Efforts to measure pO_2_ (unpublished studies) did in some cases yield a significant and positive correlation with P, with low P resulting in low pO_2_ values (an approximate drop of 20% from high to low P within the same group of pigs). This, however, was not seen in all trials, and it is likely biased by the need to capture animals for blood sampling. The CO_2_ levels, though, did increase when P levels were low. In one experiment, the pO_2_/pCO_2_ ratio was 0.51 seven days after weaning and 0.98 fifty days after weaning (unpublished data). The reason for the high CO_2_ levels is not clear. CO_2_ is known to compete with O_2_ to bind to hemoglobin and this would shift the dissociation curves shown in Figure 3 to the right, just like BPG does [28]. Hence, a question is whether animals increase their CO_2_ levels to liberate O_2_. Lactate was measured in another field study (unpublished data). It transiently increased by as much as 77% on day 26 after weaning, implying an increase in anaerobic metabolism. In the same study, CO_2_ peaked (+52%) on day 30 post-weaning, and both returned to nearly preweaning levels by day 50 after weaning (the end of the experiment), implying that the increases were not driven by metabolic activity. The piglet listed above with only 0.86 mM PO_4_ had a pCO_2_ of 9.2 kPa, thus presenting as severely hypercapnic (threshold is 6.0 kPa).

These findings tie into the above comment about sudden infant death syndrome; severe hypophosphatemia can result in suffocation despite free access to air. Moderate hypophosphatemia may well impede oxygen supply to some tissues and, through that, be responsible for other issues, e.g., ear necrosis. Field tests with P “hyper”-supplementation or with nutraceuticals that stimulate blood flow to the skin indeed have been shown to drastically reduce ear necrosis (unpublished data).

Bas Swildens (Utrecht University, Utrecht, The Netherlands), in a personal communication, mentioned that *Streptococcus suis* (Strep) requires hypoxia in the host to become pathogenic, in line with the findings of Lauer et al. [32]. This concept fits nicely with refeeding syndrome; This concept fits nicely with refeeding syndrome; discussions with technical colleagues responsible for different countries suggest that Strep appears more on the radar in countries where P levels have been scrutinized.. Farmers also report that Strep is linked to groups of pigs that are aggressive eaters. Plasma analyses confirmed that pigs that had just come down with Strep had lower plasma P levels compared to their unaffected pen mates (unpublished data). Although this discussion focuses on newly weaned pigs, in nursing infants with health issues, hypophosphatemia is also rather common [33]. Thus, hypophosphatemia may well plague nursing piglets with health challenges.

**Figure 4:**
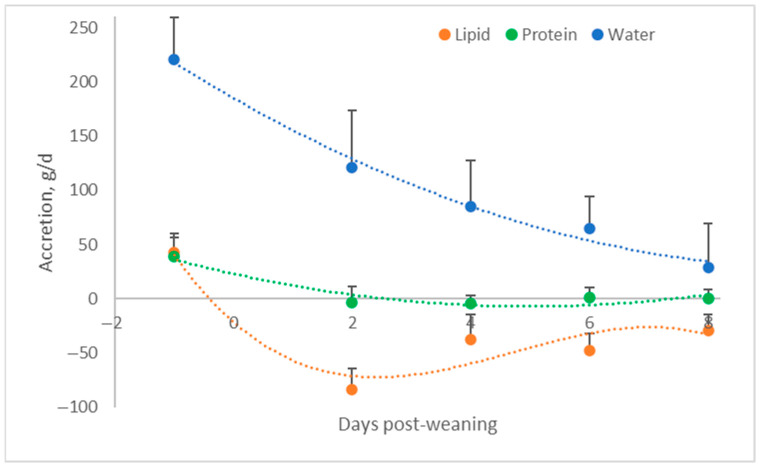
Accretion of proteins and lipids and accumulation of water around weaning (adapted from Whittemore et al. [35]).

When cells go into starvation mode, besides losing minerals, they also lose water (Figure 1). This results in a reduction in cell size. Upon refeeding, however, water is drawn into cells in amounts that exceed those prior to the fasting period. This results in edema [9,34]. This occurrence of edema was confirmed using both tissue proximate analysis and bioelectrical impedance, as described earlier [25]. It also fits with the data of Whittemore et al. [35] who showed that newly weaned pigs did not accrete protein (Figure 4). They did lose fat, though, indicating that they were eating below maintenance (and also that fat and protein accretion have different maintenance requirements) and were accreting large amounts of water, which obviously was not linked to protein gain. This water gain resulted in growth of the piglet, despite it losing fat and not gaining protein, which can explain the phenomenal feed efficiencies while eating at or below maintenance (maintenance for a 7 kg piglet is about 200–225 g of feed per day, ignoring energy expenditure resulting from stress).

## 6. A Twisty Tail

In wild animals, P deficiency can result in abnormal eating behavior. This may well be related to animals having taste receptors for P [36]. Osteophagia is a textbook example where strict herbivores start chewing on bones and carcasses. Phosphate deficiency is also listed as one of the causes of pica [37]. Pica is a compulsive eating disorder where the consumed item may or may not be food. Items commonly consumed when pica is present are things like earth, clay, or paint chips containing lead, but there are also examples of people eating their own limbs as well as cannibalism [38,39]. Zaborowski [38] states that the way to solve or prevent tail-biting is to provide calcium phosphate.

Czycholl et al. [40] and observations from farmers indicate that feed interruptions can trigger tail-biting, which again fits with phenomena of refeeding syndrome. In line with refeeding syndrome, Czycholl et al. [40] reported that almost all animals at the affected farm suffered from hypokalemia, hypomagnesemia, and hyponatremia, despite diets being adequate for those minerals. Phosphate was statistically one of the strongest affected parameters, but physiologically the differences with the controls were arguably meaningless. One caveat with this experiment is that animals were removed from the herd, transported to the research facility, and allowed to recover overnight, and only after that were blood samples taken. As explained above, this certainly perturbs feed intake, making the data for P, Mg, and K hard to interpret. Palander et al. [41] also found the highest statistical significance for P, but here, biter pigs had higher levels of blood P. One confounding factor may be that biting may become a habit even when an underlying physiological trigger is resolved. Ideally, blood samples used to identify causes of tail-biting should be collected at the earliest onset of biting and with a minimal amount of disturbance to the animals.

In some of these experiments, marker enzymes were measured. These data actually hint to rhabdomyolysis as a factor in tail-biting. Rhabdomyolysis is a muscle disorder which involves muscle tissue developing edema and breaking down. Muscle cells break open and spill their contents, which then reach the bloodstream. Myoglobin released from muscle cells subsequently damages the kidneys, which can become fatal. Symptoms include muscle pain, weakness, and tea-colored urine [42]. In humans, it is seen in a variety of conditions, ranging from accidents and exertion to nutritional deficiencies, most notably P deficiency (but Mg, K, and Na are also implicated). These nutritional deficiencies can be triggered by refeeding syndrome [43,44]. Rhabdomyolysis has also been linked to polydipsia (which is more likely to trigger hyponatremia [45]). One possible cause of muscle cell membrane disintegration is ATP depletion, which can be a result of hypophosphatemia. There is also some speculation that phospholipids on the cell surface may be sacrificed to obtain P.

Biomarkers for rhabdomyolysis include creatine kinase, lactate dehydrogenase, aldolase, alanine amino transferase, and aspartate amino transferase [42]. In a more recent publication, Lim [46] indicated that alanine aminotransferase and creatine kinase, in particular, are informative of the pathology. Interestingly, Junge et al. [47] described rhabdomyolysis in piglets two weeks after weaning, thus corresponding to the nadir in blood P as described above, and they also reported very high levels of aspartate aminotransferase, lactate dehydrogenase, and creatine phosphokinase. Czycholl et al. [40] measured creatine kinase and indeed saw a significant increase in tail-biters (+35%); aspartate aminotransferase, however, was not affected (−10%). Munsterhjelm [48] measured a more complete array of marker enzymes: creatine kinase (+36%), alanine aminotransferase (+34%), and alkaline phosphatase (+46%) increased significantly in biters, while aspartate aminotransferase was numerically increased compared to the victims (+23%). Besides the marker enzymes, there are other hints in the literature that point to rhabdomyolysis. For example, Zonderland et al. [49] reported that tail-biter pigs appeared to be experiencing pain, and they seemed to be found more often in kneeling or sitting positions, compatible with muscle issues matching rhabdomyolysis symptoms [50]. Lim [46] also lists uric acid as a biomarker for rhabdomyolysis. Urinary excretion of uric acid more than tripled shortly after weaning and returned to preweaning levels after five days (unpublished data). This theory clearly requires further study, but it fits nicely with reports that minerals containing P, Na, K, or Mg can reduce tail-biting.

## 7. Recommendations for Diet Formulation

Efforts to prevent fasting around weaning have been made but the problem still persists. A possible error that the swine industry has made in recent decades is that first-phase piglet feeds generally contain a very high glycemic load (while sow milk has a low glycemic load). Gelatinized cereals topped with sugar and highly digestible protein sources are used. This is implemented to boost palatability and, subsequently, growth. However, the gains observed do not match the expected improvement in feed intake, as fasting followed by erratic eating of high-glycemic-load feeds induces refeeding syndrome.

Indirect evidence that low-glycemic-load diets provide performance benefits across the entire nursery phase comes from a trial carried out with 900 nursery piglets. Animals were fed one of six diets that were nutritionally identical except for their starch content. Three rice starches diverse in amylose content (from 0 to 22%) were included at a quantity of 48%. Each starch was fed either raw or gelatinized (diets were fed in mash). The raw high-amylose starch had an in vitro starch digestibility of only 58%, while the zero-amylose gelatinized starch had a digestibility of 91%. Despite the much lower net energy content of the raw high-amylose diet, the piglets nevertheless had a 16% better gain-to-feed ratio and 12% higher ADG, while growth was more homogeneous [2]. Another approach for lowering the glycemic load of diets could involve an increase in the reliance on fat. Although this is technically challenging, it may also improve palatability [51]. Fibers present yet another possibility, but with the challenge that fibers require adaptation of the microflora; this adaptation period arguably surpasses the period during which the animals develop refeeding syndrome.

When discussing this issue with commercial nutritionists in both the USA and Europe, we found that there are indeed companies that have developed piglet feeds that follow a different principle; their diets are not stuffed with gelatinized starch and sugar, and thus, they have a low glycemic index. Piglets that were fed those diets grew poorly after weaning but in line with what would be expected based on energy intake. Long-term measurements, though, apparently indicate that they grow better overall.

One unresolved question is the impact of refeeding syndrome on bone development. Blood data show that P levels can remain below targets for nearly the entire nursery period, meaning that the piglets increase in size by a factor of 3 to 4, while bone development may lag behind. Phosphate requirements for phase I nursery feeds, as used in academia and industry, appear to be foremost extrapolated from older pigs without any consideration for what the actual feed intake levels, eating patterns, or glycemic loads are, let alone considering any effects of stress. Cemin et al. [52] performed two large-scale dose–response studies which showed that mortality and removals were lowest at 0.52% available P (16.4% at 0.33% vs. 9.7% at 0.52% available P). Cemin et al. [52] calculated the requirements using regression of 0.59% available P. In line with this, lysine requirements for newly weaned pigs cannot be 1.2% or more if the animals are eating at maintenance, as this results in zero protein accretion. Studies in which plasma amino acids were measured indeed show that plasma lysine increases after weaning, while amino acids that are key for managing oxidative stress (e.g., cysteine, histidine) decrease to practically zero (unpublished data). Arguably, the entire nutritional profile of a diet for newly weaned piglets should be dictated by their real feed intake and the health challenges they face, such as refeeding syndrome, rather than to support muscle gain.

## 8. Conclusions

Newly weaned pigs commonly develop hypophosphatemia as a result of refeeding syndrome, resulting in edema. This edema is not concentrated in the belly, as in Kwashiorkor [53], for example, but rather, it is in other tissues such as muscles, possibly due to rhabdomyolysis. Due to this edema, newly weaned pigs appear to be growing when, in fact, they are simply retaining water. A simple energy requirement calculation shows that this problem is widespread in nursery trials; despite eating around maintenance levels, newly weaned piglets in just about all published studies gained weight. Refeeding syndrome should be considered as a factor in weaning stress, and arguably, historic data should be re-interpreted considering that high feed efficiencies shortly after weaning are a sign of problems, not a sign of success.

## Data Availability

Not applicable.
